# Patients and Medical Staff Attitudes Toward the Future Inclusion of eHealth in Tuberculosis Management: Perspectives From Six Countries Evaluated using a Qualitative Framework

**DOI:** 10.2196/18156

**Published:** 2020-11-02

**Authors:** Ioana Margineanu, Christina Louka, Maria Vincenti-Gonzalez, Antonia Morita Iswari Saktiawati, Johannes Schierle, Kabiru Mohammed Abass, Onno Akkerman, Jan-Willem Alffenaar, Adelita V Ranchor, Ymkje Stienstra

**Affiliations:** 1 Department of Clinical Pharmacy and Pharmacology University Medical Centrum Groningen University of Groningen Groningen Netherlands; 2 Pneumology Hospital Iasi Iasi Romania; 3 Department of Internal Medicine University Medical Centrum Groningen University of Groningen Groningen Netherlands; 4 Department of Medical Microbiology and Infection Prevention University Medical Centrum Groningen University of Groningen Groningen Netherlands; 5 Department of Internal Medicine Faculty of Medicine, Public Health and Nursing Universitas Gadjah Mada Yogyakarta Indonesia; 6 University Medical Centrum Groningen University of Groningen Groningen Netherlands; 7 Agogo Presbyterian Hospital Agogo Ghana; 8 Department of Pulmonary Diseases and Tuberculosis, Tuberculosis Centrum Beatrixoord University Medical Centrum Groningen University of Groningen Haren Netherlands; 9 University of Sydney Camperdown Australia; 10 Westmead Hospital Sydney Australia; 11 Marie Bashir Institute of Infectious Diseases, University of Sydney Sydney Australia; 12 Health Psychology Section University Medical Centrum Groningen University of Groningen Groningen Netherlands

**Keywords:** eHealth, tuberculosis, policy, clinical, patient, perspective

## Abstract

**Background:**

Digitally delivering healthcare services is very attractive for tuberculosis (TB) management as this disease has a complex diagnosis and lengthy management and involves multiple medical and nonmedical specialists. Especially in low- and middle-income countries, eHealth could potentially offer cost-effective solutions to bridge financial, social, time, and distance challenges.

**Objective:**

The goal of the research is to understand what would make eHealth globally applicable and gain insight into different TB situations, opportunities, and challenges.

**Methods:**

We performed focus group interviews with TB experts and patients from 6 different countries on 4 different continents. The focus group interviews followed the theory of planned behavior framework to offer structured recommendations for a versatile eHealth solution. The focus group interviews were preceded by a general demographic and technology use questionnaire. Questionnaire results were analyzed using basic statistics in Excel (Microsoft Corporation). Focus group interview data were analyzed using ATLAS.ti 8 (ATLAS.ti Scientific Software Development GmbH) by assigning codes to quotations and grouping codes into the 5 domains within the framework.

**Results:**

A total of 29 patients and 32 medical staff members were included in our study. All medical staff had used the internet, whereas 31% (9/61) of patients had never been online. The codes with the most quotations were information in relation to eHealth (144 quotations) and communication (67 quotations). The consensus among all participants from all countries is that there are important communication and information gaps that could be bridged by an eHealth app. Participants from different countries also highlighted different challenges, such as a majority of asylum-seeker patients or lack of infrastructure that could be addressed with an eHealth app.

**Conclusions:**

Within the 6 countries interviewed, there is high enthusiasm toward eHealth in TB. A potential app could first target information and communication gaps in TB, with additional modules aimed at setting-specific challenges.

## Introduction

The continuous growth of the internet and availability of smart technologies have modified many aspects of life, including health care delivery. With more than half the world’s population online [1], delivering health-related services digitally has never been more appealing or accessible, with the eHealth market expected growth estimated at 22% by 2024 [2].

Tuberculosis (TB) is one the deadliest infectious diseases worldwide, with an estimated 1.5 million deaths yearly [3]. The more resistant forms, like multidrug-resistant TB and extensively drug-resistant TB, pose a new threat, with treatment success rates between 50% to 60% globally. TB diagnosis is complex, its treatment is lengthy, and it requires close collaboration of different medical and nonmedical experts and patients to ensure TB management is adequately performed, especially in challenging settings such as patients living in remote locations or constrained by socioeconomic factors.

Studies piloting various types of eHealth technologies conducted around the world have evaluated multiple areas where eHealth could aid in TB management. From improving communication among medical staff [4] and patients [5] to reducing costs [6] and improving treatment indicators, especially in situations where patients were traveling [7] or in hard to reach regions [8], eHealth seems to be a promising field of research and a useful, cost-efficient, and acceptable improvement for TB management.

On the other hand, multiple studies and reports, including a consumer report from the European Union [9], have observed that there are certain difficulties when implementing eHealth, such as lack of acceptance, unfavorable regulations, and insufficient funding [10]. The progress of eHealth in lower income countries is limited by lack of know-how, funding, technology, and communications ability [11].

Taking into consideration the particular nature of TB and population differences together with the potential benefits and challenges of eHealth, tailored research would aid in creating useful, tailor-made eHealth apps. This idea is recommended in multiple studies, including a recent review of smartphone apps [12]. Market research, defined as an “organized effort to gather information about target markets” [13], is an important component of any business strategy aiming to create a new product. In order to make sure a product is useful, acceptable, and qualitative, market research is performed to understand the needs and preferences of the market. One of the frameworks used to conduct market research is the theory of planned behavior [14], which links psychological intent to three determinants: attitude toward behavior, subjective norm, and perceived behavioral control. This theory attempts to explain behavioral intentions and has been previously used in numerous studies to investigate and explain intention and possible adoption rates of new interventions [15,16].

This study aims to identify potential user perceptions about eHealth use in TB management. We used the theory of planned behavior framework to interview TB experts and patients in 6 diverse countries on 4 different continents to form recommendations for a well-received, usable, comprehensive, and efficient TB eHealth app.

## Methods

### Participants

Adult (over aged 18 years) participants from Romania, Greece, Netherlands, Indonesia, Ghana, and Venezuela were approached in the collaborating clinics and invited to participate. Two participant groups per country were interviewed, one comprising actual or former TB patients and one of the TB medical staff experts. Experts had to have worked in a TB clinic on a daily basis for at least 3 years. Participants were recruited through purposive sampling by investigators visiting different medical facilities specializing in TB care. The target size was 2 to 6 participants per focus group as all participants were highly involved with the topic of TB and discussion in larger groups was not recommended [17].

### Study Design

The study was performed in two parts. The first phase consisted of a short questionnaire to collect demographic data and basic internet and mobile use statistics by asking the experience, in years and number of hours per day, spent using the internet and smartphones and a numbered scale on which participants ordered activities performed in order of frequency (with 5 being most frequent and 1 least frequent). Activities were defined as communication (eg, WhatsApp, Facebook Messenger), social media (eg, Facebook, Instagram), utilities (eg, banking, weather), work, games, and health/medical.

The second part consisted of semistructured focus group interviews with questions as conversation starters. The researchers allowed the interviewees to have a conversation around a specific question and asked follow-up questions for clarification. Questions were designed to repeatedly ask the same subject in different ways and at different time points during the interview to achieve data saturation irrespective of time needed. In order to adhere to qualitative reporting standards, the consolidated criteria for reporting qualitative research (COREQ) checklist was used [18].

In order to minimize the risk of bias, the researcher conducting the interviews had no previous history with the patients or medical staff involved and conducted the interviews in a private room, patients separated from medical staff. Interviews were conducted in the participants’ native tongues or in a language they felt comfortable in, transcribed verbatim, and translated by a native speaker with proficiency in English.

### Framework

In order to develop a globally acceptable app that can be used in different settings, interviews targeted diverse countries with different cultures, socioeconomic statuses, and TB populations. Thus, 6 countries, Romania, Ghana, Indonesia, Greece, the Netherlands, and Venezuela, were chosen to have a mix of geography, socioeconomic statuses, health care systems, and TB profiles (Multimedia Appendix 1).

The focus groups followed a semistructured framework based on the theory of planned behavior (Textbox 1). This psychological theory proposes that intention—in our case, use of eHealth—has a number of determinants. The original work describes three main determinants, and these were used in order not only to stay true to the framework, but also to guide interviews in a simple, efficient manner. The first is the attitude toward the behavior, defined by the strength of the attitude and the evaluation of the outcome, favorable or unfavorable. The second is the subjective norm, or beliefs about the normative expectations of others: perceived social pressure to perform or preclude from the behavior. The last is the perceived ease or difficulty of performing the behavior and is based on past experience as well as anticipated factors that might facilitate or impede a specific behavior [14]. The last domain, preferred features, was added to further stratify user preferences.

**Interview structure based on the theory of planned behavior.**

Attitude toward behavior:

- Q1: Which problems and challenges in tuberculosis management could be solvable by an eHealth solution?
- Q1 follow-up: If you had to identify priorities, which would be the biggest challenge?

Subjective norm:

- Q2: How do you think the medical staff and patients here would react to the implementation of an eHealth solution?
- Q2 follow-up: What do you think will be the biggest problem or motivator to accept and use eHealth?

Perceived ease or difficulty of adopting behavior:

- Q3: Do you use technology regularly to assist with your work or patient life (for admitted patients)?
- Q3 follow-up A: Do you think the implementation of various software solutions has made your life (work/patient) easier or harder?
- Q3 follow-up B: Why?
- Q3 follow-up C: What could be done to a new eHealth app to make it really useful?
- Q4: Do you think implementing eHealth in your daily lives will be easy or difficult?
- Q4 follow-up A: Why?
- Q4 follow-up B: What would make it easier or harder to implement?

Preferred features:

- Q5: Name 5 features or things you would likely use most in an eHealth app.
- Q5 follow-up: Which one do you feel you need most? Which process of tuberculosis management would be most suitable to be streamlined through eHealth?

### Ethics

The study was approved by the ethics committee of the initiating institute, the University Medical Centrum Groningen (METc 2017/448), and the medical facilities of each country participating in the study. All focus group participants signed informed consent expressing their volition to participate in the study.

### Data Collection and Analysis

A thematic approach using ATLAS.ti 8 (ATLAS.ti Scientific Software Development GmbH) was used to analyze the transcripts by one investigator (IM); this was reviewed by the supervisory authors. After major themes were identified in response to the questions asked and the theory of planned behavior framework, the transcriptions were indexed using topic coding (Textbox 2). Codes were assigned to phrases addressing an issue in the positive or negative (eg, “Maybe if we use an app, [the process] would be made simpler” versus “[eHealth] would give us double the job”) and were assigned whenever a quote was repeated by other participants in the focus group but not by the same participant. For clarification purposes, see definitions in Multimedia Appendix 2. All of the coding was performed by two independent authors (IM, CL). Conflicts were resolved by discussions between the coders with the aid of one of the supervisors (YS).

Descriptive statistics were used to present quantitative results. Medians and interquartile ranges were used to present internet and mobile experience. Codes were reported as a total and per domain, and themes were reported as proportions of the total.

**Coding.**

Attitude toward behavior:

- communication – eHealth
- eHealth – speed
- eHealth – would/wouldn’t help in tuberculosis
- information/education – eHealth
- my own experience with tech

Subjective norm:

- communication – status quo
- how will others react to eHealth in tuberculosis
- information – status quo
- local environment
- others’ experiences with tech
- stigma/isolation

Perceived ease/difficulty of adopting behavior:

- Community building/testimonials
- I can/I can’t implement eHealth
- Localization
- Money/devices
- Privacy/confidentiality
- Repeatability
- User interface/simplicity
- Training
- Videos

Preferred features:

- Screening/prevention
- Diagnosis
- Treatment
- Reminders
- Gamification
- Media preference
- Anything else

## Results

### General Questionnaire Results

A total of 29 patients and 32 medical staff members responded to the questionnaire and participated in the focus group. Four patients were former TB patients and had finished their treatment within 6 months of the interview, with the majority being current patients. Ages ranged between 23 and 63 years for patients and 30 and 60 years for medical staff. Gender distribution was mostly male (20/29, 68%) among patients and mostly female (26/32, 81%) among medical staff; 65% (19/29) of patients and 68% (22/32) of medical staff lived in urban environments. Education levels were lower in patients groups than in the medical staff group: among patients, the highest level of education was a bachelor’s degree, and among medical staff, the highest level was doctorate (PhD).

**Figure 1:**
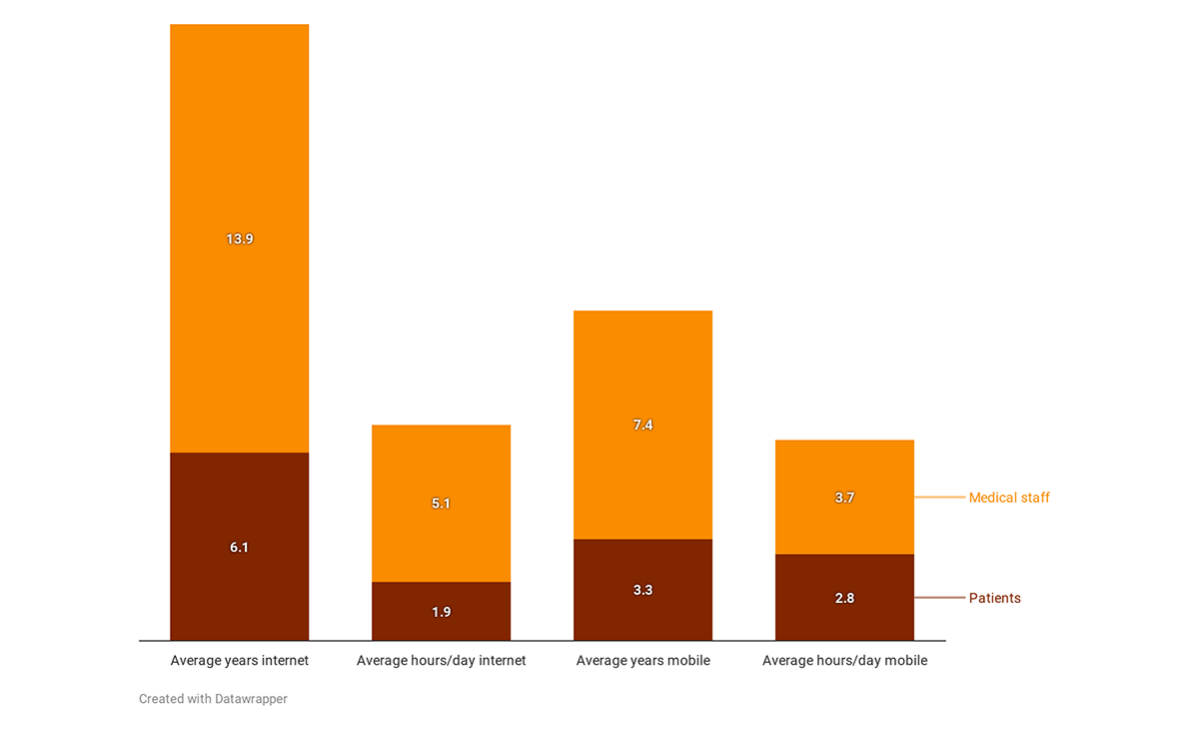
Internet and mobile experience for the two participant groups.

General internet and mobile internet use was almost half within the patient group compared with the medical staff (Figure 1): average years of experience using the internet was 6.1 (range 1-16) person-years versus 13.9 (range 5-35) person-years. All medical staff had used the internet, whereas 31% (9/29) of patients had never been online. Concerning mobile phone use, staff median experience was 7.4 (range 0.3-15) person-years versus 3.4 (range 0-10) person-years for patients; 44% (13/29) of patients had never used mobile data and 34% (10/29) had never used a smartphone. Of all patient groups, the least tech savvy were the Romanians and the most were from Venezuela. From the medical staff groups, participants from Ghana had the least experience with the internet and mobile, and participants from the Netherlands had the most. Internet and mobile use in hours per day were lower for people from the low- and middle-income countries than the high-income countries, but this correlation was observed only in the patient groups and not in the expert groups.

The 20 patients who used the internet mostly used it for communication (median 4/6), followed by social media (median 4/6), utilities (median 3/6), medical/health (median 2/6), work (median 1/6), and games (median 1/6). For smartphones, the 18 patients ordered the categories the same, but with more patients using Messenger (median 4.5/6) and fewer using for social media and utilities (median 3/6). The 32 staff members used the internet mostly for communication and work (median 5/6); games were the least used (median 1/5). Medical staff use smartphones also mostly for communication (median 5/6), followed by social media (median 4/6), and work (4/6).

### Focus Group Interviews

The final yield was 13 focus group interviews, two per country, with 2 focus groups conducted with Dutch medical staff as the TB clinic and at the Municipal Health Center Groningen (a type of outpatient clinic) are geographically separated. The duration of interviews ranged between 25 and 45 minutes.

By domain, attitude toward behavior contained the most quotes (339), with preferred features containing the least (161). By far the most discussed topic was the potential of eHealth to improve information and/or education concerning TB among patients, the public, and even medical staff (144 quotes). The countries that mentioned this aspect the most were Venezuela (55 quotes) and the Netherlands (31 quotes).

A summary of the most frequent themes/codes and example quotations can be found in Multimedia Appendix 2.

### Thematic and Code Analysis

The attitude toward behavior domain contained 5 codes, with a total yield of 339 quotations out of the total 911 quotations (37.2%), the most of all domains. Even though experience varied between patients and medical staff, both groups explained that their outlook concerning implementing eHealth in TB was overwhelmingly positive (292/339, 86.1%), with the most expected impact in the information and communication fields.

Education [would be the first priority when implementing eHealth].

Medical staff, Romania

Actually there is already a program for TB managing. It has been running actually, but not everyone used it [referring to an electronic database for medical staff].

Medical staff, Indonesia

Maybe if using an app, would make it simpler.

Medical staff, Indonesia

Participants also offered examples of specific ways in which eHealth could improve TB management.

It’s important to really explain that the disease is treatable and to know more about this disease and to say that people can survive.

Patient, Venezuela

Participants felt that speed, convenience, flexibility, and the ability to communicate and obtain information over long distances were very important factors contributing to a positive outcome.

And also the plain fact that they wouldn’t have to come to the hospital could be a motive [to adopt an eHealth app].

Medical staff, Greece

Although the expected outcome is mostly positive, some challenges have been identified, such as a need for personal contact. Interestingly, within the same focus group, one person believed an app would enhance contact and others did not.

The fault is the lack of contact in DOT and an app could do that.

Medical staff, the Netherlands

Yes, but nothing can replace the live contact.

Medical staff, the Netherlands

Furthermore, patients mentioned a need for trustworthy sources of information, and some medical staff were concerned about patients intruding on their personal time if the app were to be on their personal phones or of doubling the workload (traditional system and eHealth).

The subjective norm domain contained 6 codes with 22.8% (208/911) of the total quotations. All groups felt there is a certain degree of pressure to perform the behavior as they felt the status quo should be improved, and they thought eHealth could be a useful tool for such improvement. Participants described gaps especially in communication and information access that would encourage them to use a trusted eHealth app.

There needs to be more explanations about what you can do, more explanations about the multiple versions of TB. We don’t know about that you can have it in your bones, people don’t know about adverse reactions, we don’t know about these things.

Patient, the Netherlands

Sometimes it [the internet search] will not give you a straight answer. Sometimes it will lead you to further look.

Patient, Ghana

Concerning the particular use of eHealth in TB and the perceived subjective norm concerning the general public, patients spoke about stigma and how eHealth, through education, could solve such problems.

For example, I feel a lot of rejection every time; people changed a lot when they found I am TB positive, they don’t even say hello directly to me, but from afar and this rejection is due to the lack of knowledge, education.

Patient, Venezuela

Participants believed either that eHealth would be easy to implement or that it is possible but that ease of implementation will depend on certain factors, most frequently technology savviness.

If I could use technology, I would use it. But now I am afraid I would make mistakes whilst using it because I would get confused.

Patient, Greece

Our TB patients are not there yet. We don’t really have university professors with TB.

Medical staff, Romania

Participants also mentioned lack of resources as possible impediments to eHealth implementation. Lower income countries tended to mention more often a lack of physical resources, such as electricity, network coverage, or hardware (computers, phones), whereas higher income countries were worried about the lack of human resources needed to manage a potential extra burden of eHealth.

They [the patients] get their phones stolen, or all sorts of cables can be robbed.

Medical staff, Venezuela

The [clinic] does not have the manpower to see everyone, they [clinic staff] go [to see patients] maybe once every two months.

Medical staff, the Netherlands

Perceived ease or difficulty toward behavior contained 9 codes and 22.2% (203/911) of the total quotations. A minority of participants expressed they didn’t think they could use a new app, quoting reasons such as illiteracy or lack of time. Most either said they would have no problems integrating a new app within their digital routines or that they would require certain facilitators that would promote adoption (eg, have a simple interface, contain a training module).

We are always open to new technology which could help us improve even more.

Medical staff, Greece

[Asked how they would respond to new app] Easy, easy [raised voice, altogether].

Patients, Venezuela

And if you have too many apps, like we already do, to have another one, it could be time consuming. We wouldn’t want to be overloaded.

Medical staff, the Netherlands

Participants identified steps that are mandatory to perform in order to have an easy-to-use app, such as privacy or localizing the app.

Privacy, that’s what I would consider first. There shouldn’t be any breaches because otherwise it wouldn’t succeed so no one should have access to data.

Patient, Greece

Facilitators concerning implementation, such as financial incentives in the form of extra staff or equipment or offering the app for free were also identified, especially since some participants expressly mentioned the financial difficulties some patients are in.

I believe if the app could be in every language and the devices would be provided people would be very grateful, so it won’t also be useful, but they would be more compliant.

Medical staff, Greece

Then the cost. It should be free.

Patient, Ghana

Some participants expressed a desire to be trained in how to use the app or for a demo module to be presented as they believe this would facilitate quick adoption.

Some of us will need someone to teach us.

Patient, Romania

Concerning the app itself, participants believed ease of use would be furthered by a simple and friendly user interface, by adding a community-building module (either for the patients or for medical staff), adding a training module, and using videos to transmit information.

It should be simple, with not many things, because it would demotivate me.

Medical staff, the Netherlands

It can also even have people who are also being successfully treated, people like that communicating, all that being put into the app so that when the person goes, person knows from the beginning how the treatment goes, and after treatment what to expect.

Medical staff, Ghana

Preferred features contained 7 codes with 17.6% (161/911) of the total quotations. Concerning information media, most participants expressed a preference for video, followed by images, text, and a gamification component, such as quizzes, to enhance the user experience.

I think film would be nice. When you read you can put it away.

Medical staff, the Netherlands

Or maybe a countdown, how far you are and how much you have left and then patients know what to eat. For them to see how easy it is and to stimulate them to continue.

Medical staff, the Netherlands

Participants would rather use the app for treatment (38/61) than for diagnosis (17/61) or prevention (19/61), and they would want a notification or reminding system implemented to encourage treatment adherence and follow-up.

Patients can use an application to schedule visits to health facilities, start treatment, when should do sputum test, do monthly check up. Things that they usually do here, but it will be paperless/electronically. Then all these [digital] notes can be brought everywhere, I mean paper note can be lost, can be damaged.

Medical staff, Indonesia

Through this media/app patients can be educated and we can reinforce prevention.

Medical staff, Venezuela

## Discussion

### Principal Findings

Using a health behavior framework for market research [19], this study explored attitudes toward eHealth implementation in TB. Semistructured focus group interviews were performed worldwide with medical staff and patients to better understand key motivators, challenges, facilitators, and user preferences for implementing new eHealth solutions. A number of important insights have been gathered, as has a prioritization of features to be implemented, which can be used when planning new eHealth apps for TB. Overall, both patients and TB experts have expressed enthusiasm at the potential of eHealth, with an overwhelming consensus that the first domains where it could be useful are information and communication.

The attitude toward eHealth domain contained the most codes. We interpreted this result in the context of participants having already formed an opinion on this subject and welcomed the opportunity to discuss it in depth. The domain presents encouraging results, with 67% (229/339) of codes expressing a positive expectation about eHealth capabilities. Participants overwhelmingly felt there is a lack of knowledge about TB among patients, the general public, and even among medical practitioners. These findings are mirrored by a systematic review that concluded there is a lack of knowledge among medical practitioners concerning national or internal TB guidelines within 14 non-European countries [20] and by multiple studies identifying knowledge gaps among patients [21-23]. Furthermore, a recent review highlighted that many apps offer inaccurate information [24]. Participants expect that an eHealth solution would be used to educate and provide accurate, secure, and friendly information.

The subjective norm describes the most important pressure to adopt eHealth as the lack of information and communication, felt across the board. Participants described a lack of clear, open communication channels both between patients and medical staff and between different specialties involved in TB management. Multiple studies have linked lack of patient-medical staff relationships to poorer outcomes in TB [25-27], concluded that communication methods should be tailored [28], or called for improving collaborations between medical staff involved in TB care [29]. Participants in our study would not only welcome online TB-related communities but also believe that communication could be improved through an app and that, in itself, would improve TB management and the stigma felt at the moment. On the other hand, a challenge identified was the need for human, personal contact, identified especially by a minority of Dutch participants (2).

Concerning perceived ease or difficulty of use, most participants felt they could implement a new app easily, although some participants mentioned a fear that a new app would be time consuming.

One important factor that could influence adoption was identified as technology savviness, linked to age and experience with use; however, some participants felt that training could bridge this gap. Indeed, one study using video directly observed therapy noted that “Older participants in particular enjoyed learning to use a smartphone” [30]. A facilitator mentioned by some participants is localization, translating the app in the local language. A minority of participants quoted illiteracy as a barrier to regular app use.

On a local level, interviewees from countries with a resource paucity, such as Venezuela or Greece, expressed a need for extra human or physical resources. Most participants agreed that an app should be free. Interestingly, concerning technology accessibility, opinions varied widely, from “even if they are illiterate they use the internet, google, even if they can’t write their own signature, they can go online” to “they probably have 1 smartphone per family and they don’t use the internet all the time.”

From a development perspective, the only truly mandatory feature to be implemented would be privacy/security, as this was a concern expressed in multiple interviews, both by experts and patients. A recent systematic review performed by the authors (unpublished) highlighted that within 7 studies that quantified this aspect, there were zero privacy breaches for a pooled 71 patients.

Participants mentioned other features, such as a treatment module with asynchronous video therapy, reminders, and a diagnosis module with an emphasis on self-diagnosing education in order to hasten hospital visits. Participants also expressed a desire to have a gamification component and believed video would best facilitate app adoption.

Textbox 3 summarizes recommendations for developing a new eHealth app for TB.

**Recommendations for developing a new eHeath app for tuberculosis.**

General:

- Target the app for education, followed by communication, treatment, prevention, and diagnosis
- Identify a multidisciplinary team that can create a universally usable app, but recruit local members to advise, tailor-make, and translate
- Create a concept revolving around modules or build separate apps (eg, for medical staff and for patients)

App-related:

- Make sure the app is private and secure
- Focus on a simple and friendly user interface with a preference for images/icons over text
- Create training and educational videos
- Offer the app for free

Make sure the app is private and secure:

- Use an institutional server if your institution already has taken security measures for its existing patients
- Use an external secure server for additional privacy
- Respect local and regional privacy laws (eg, General Data Protection Regulation in the European Union)

Implementation:

- Find local tech leaders who can learn the app quickly and facilitate dissemination and further education
- Preferably, offer devices for at least the most disadvantaged members of the target populace
- If designing a medical staff app, discuss with administrators so that the app decreases the workload and doesn’t add to it
- Have an active tech support

Modules:

- Education module, preferably with a gamification component
- Communication components:
  medical staff (results, expert forum)
  patients (testimonials, community)
  medical staff and patients (side effects, questions, and appointments)
- Treatment module: calendar, video/directly observed therapy, reminders

### Comparison With Prior Work

There are few studies exploring user attitudes for eHealth in TB. A study from Mozambique exploring text messaging found, as our study did, that messaging should be used for reminders and motivational texts in order to increase retention and that the main obstacle would be privacy assurance [31]. Another study involving focus group interviews with medical staff for a chronic obstructive pulmonary disease telerehabilitation app found that education and skill training are highly essential to support successful implementation [32]. A study from Saudi Arabia showed that perceived usefulness and perceived ease are significant factors to performing the behavior, results corroborated by our study [33]. Furthermore, a recent systematic review (unpublished) performed by the main authors revealed that already implemented eHealth apps focused rarely on education, despite it being one of the two major needs felt by the participants within the focus groups.

This study offers a more diverse perspective on eHealth use in TB by conducting interviews in 6 countries on 4 different continents to gain a more global perspective for a potential app that could be universally applicable.

### Limitations

Purposive sampling was used, which might have selected participants already more open to new technology. Furthermore, individual interviews might have elicited different results as they have less risk of bias and are more in-depth. The theory of planned behavior is a useful tool to gauge decision making with the caveat that it does not take in account socioeconomic, religious, and gender-based factors.

### Strengths

This study was conducted in 6 different settings, offering a better understanding of what populations across the globe might decide concerning adopting eHealth in TB management. TB patients and TB experts were approached, thus covering the potential user base for future eHealth solutions. The theory of planned behavior is a simple, elegant way to conduct focus group interviews and understand decision-making processes. Two independent authors coded the interviews, thus limiting bias.

### Conclusion

This study used focus group interviews performed in 6 countries in order to gauge perceptions about eHealth use in TB management and draw recommendations for further implementation. Participants in all 6 countries are enthusiastic about eHealth, and most users expect a potential app to be helpful. There is a global need to improve information access and communication, and participants feel that eHealth could help bridge this gap. Most themes resounded in all countries interviewed, but certain particularities, such as a large proportion of asylum seekers or lack of infrastructure or training, should be addressed when trying to implement eHealth in specific settings. Despite individual preferences, the global sentiment is that eHealth is a promising field of research that will be well received with the potential to enhance multiple aspects of TB care, with an emphasis on the need to communicate and educate.

## Appendix

Multimedia Appendix 1 Country profiles.

Multimedia Appendix 2 Definitions.

Multimedia Appendix 3 Themes and codes ordered by frequency.

## Abbreviations

COREQ: consolidated criteria for reporting qualitative research
TB: tuberculosis
