# Supplementary material for: Patients and Medical Staff Attitudes Toward the Future Inclusion of eHealth in Tuberculosis Management: Perspectives From Six Countries Evaluated using a Qualitative Framework
Source: JMIR Mhealth Uhealth. 2020 Nov 2;8(11):e18156. doi: 10.2196/18156 (PMC7669445; doi:10.2196/18156)
Supplement: Multimedia Appendix 1 [file mhealth_v8i11e18156_app1.docx]

Multimedia Appendix 1. Country profiles.

| Country | Income | TB incidence | TB particularity | Continent |
| --- | --- | --- | --- | --- |
| Romania | Medium | 74:100.000 | Within the EU, Romania has still very high TB incidence which accounts for 23.1% of EU TB. MDR and XDR numbers have increased in recent years. | Europe |
| Greece | High | 4.5:100.000 | Greece is a low incidence country which has received additional TB cases through migration. The financial crisis complicated TB management. | Europe |
| Netherlands | High | 5.2:100.000 | The Netherlands has successful treatment outcomes in TB because of a standardised national response. They are a low-incidence country where most TB cases are discovered within foreign-born nationals. | Europe |
| Indonesia | Lower-middle | 391:100.00 | TB is under notified and 1 out of 2 treatment plans are inappropriate thus resistant TB is a major issue. | Asia |
| Ghana | Lower-middle | 156:100.000 | Ghana is struggling with a lack of resources to tackle TB and a large number of HIV+ persons. HIV+ among TB patients varies between different geographical regions in Ghana, ranging between 9.4% and 33.4%. | Africa |
| Venezuela | Low | 34:40.0 | With advent of the financial crisis, Venezuela, a country which used to have low incidence, how has seen a resurgence of TB cases. Lack of resources impedes TB management. | South America |
