# Supplementary material for: Patients and Medical Staff Attitudes Toward the Future Inclusion of eHealth in Tuberculosis Management: Perspectives From Six Countries Evaluated using a Qualitative Framework
Source: JMIR Mhealth Uhealth. 2020 Nov 2;8(11):e18156. doi: 10.2196/18156 (PMC7669445; doi:10.2196/18156)
Supplement: Multimedia Appendix 2 [file mhealth_v8i11e18156_app2.docx]

Multimedia Appendix 2. Definitions.

| - **Domain** – one of the main four code groups used, eg, “Subjective Norm”.   - **Code** – topic of conversation. Can be supportive or against a certain common idea, eg, “eHealth would be time efficient” and “eHealth would make us work doubly” both belong to the same code     - **Theme** – within a code, a positive OR a negative attitude towards a code, eg, “eHealth would be time efficient” and “eHealth would make us work doubly” are two opposing themes within the same code       - **Quote** – direct quote from a focus group participant. |
| --- |
