# Supplementary material for: Patients and Medical Staff Attitudes Toward the Future Inclusion of eHealth in Tuberculosis Management: Perspectives From Six Countries Evaluated using a Qualitative Framework
Source: JMIR Mhealth Uhealth. 2020 Nov 2;8(11):e18156. doi: 10.2196/18156 (PMC7669445; doi:10.2196/18156)
Supplement: Multimedia Appendix 3 [file mhealth_v8i11e18156_app3.docx]

Multimedia Appendix 3. Themes and codes ordered by frequency.

| Domain: Attitude Towards Outcome  *What do I expect to happen when implementing an eHealth app? What do I expect it will be useful for?* | |
| --- | --- |
| **Code: Information/education in relation to eHealth, n=144** | |
| Key themes | Examples |
| eHealth would help for information or education, especially in this particular ways/settings, n=96 | “The app should send to people what could happen to them.” FG3  “There needs to be more explanations about what you can do.” FG4  “It’s important to really explain that the disease is treatable and to know more about this disease and to say that people can survive.” FG7  “So educative sessions which are really up to date because to address the ignorance from the patients. Topics like transmission and ways of transmission.” FG8  “I think that it would probably give people who are not even health workers a broad view about the whole condition. Because if you are there to know about the symptoms and the presentation from the app then you easily know that, I should probably be thinking of this very condition and therefore need to go to the hospital for check- up.” FG13 |
| Information/Education should be the first priority when designing an app, n=40 | “Education. Mentality changes with social interaction.” FG6  “Mainly focused on education.” FG8  “Yea. For more information.” FG12 |
| There are some challenges/ I am sceptical about certain aspects concerning using eHealth to inform, n=8 | “No, because people are prejudiced and I don’t think this would help because it might scare them more to read about that stuff” FG1  “One of the challenges would be that the app is known as trustworthy, so we can know the app is related to the formal sources, so with the ministry of health.” FG7 |
| **Code: Communication in relation to eHealth, n=67** | |
| Key themes | Examples |
| eHealth can be used for specific types/settings of communication, n=17 | “Video if they relocate, for example in another country.” FG9  “The app should contain a system where each person can create their own user and be able to contact the experts for medical doctors in rural areas or resident doctors who want to contact the experts…through this app this should be possible” FG8  “And the also you can include the management of the symptoms of the condition aside from using the medication and the treatment, I mean management that is convenient at home.” FG13 |
| eHealth can facilitate patient - doctor communication, n=20 | “Yes that [using an app for communication] would be perfect because if I cannot communicate with my doctor, I will go only and I will find all this different information and I don’t know which is correct and wrong” FG1  “But you have to find a way to reach and communicate with as many patients as possible.” FG2  “I also think that application’s purposes are just sharing knowledge and information. Only for consultation, and not giving prescription or medicine.” FG10  “Interviewer: Of all these things, which is most important to you? Which is number 1?  FG Participant: That there is a specialist [at the other end of the app].”FG12f |
| There are certain obstacles in using eHealth for communication, n=11 | “But you can't do that with an app, obtain the homely contact.” FG3  “So the more difficult thing is the globalisation of the app to enable the communication between peers and doctors like inside one country and between countries, this would be the most difficult.” FG8  “If they couldn’t reach us with our personal phone then we would use the app.” FG2 |
| eHealth can facilitate doctor-doctor communication, n=11 | “How the report[s] can be done with discipline and in order… because [they] involve many parties like the 5th floor staffs, doctors, pharmacist. In order to create a more integrated system for the health providers.” FG11  “So to start contacting other peers, other specialists, to build up the information which will be managed among medical personnel and to also contact people and specialists from other countries.” FG8 |
| eHealth will make communication easier, n=9 | “And if [in our country] it would be working as it should it could enhance our quality of life in terms of communication.” FG8  “Automatically the application can be accessed and used from anywhere.” FG11  “Yes, I would love it. Here, we talk all the time. I think it would be very useful.” FG5 |
| **Code: My own experience with technology, n=58** | |
| Key themes | Examples |
| Based on my experience with technology, I will probably use an app/ have little difficulty adopting a new app/ I won’t feel pressured or afraid to use a new app, n=40 | “My opinion is that the internet is this century’s best discovery.” FG6  “It’s so easy.” FG9  “Well apps are designed for easy use, I don’t think I had an app I did not know how to use…” FG9  “Yes apps and technology make our lives easier for example with bank transfers to purchase things I think it’s important.” FG7 |
| Based on my experience, I will find it difficult to adopt a new app/ I am afraid to use technology/ Technology makes me feel uncomfortable, n=18 | “The best example is when the power goes out. You feel a complete lack of control. It’s the same with computers.” FG6  “I am illiterate so I wouldn’t be able to use the app.” FG1  “No [the internet is indeed not secure] because for example people use internet to gossip and u can explore the private life of others” FG11 |
| **Code: eHealth would/would not help in TB, n=42** | |
| Key themes | Examples |
| An app would help in TB management, n=33 | “I will support health application development. Please publish it as soon as possible.” FG10  “Yes of course it would make it easier.” FG1  “But it would help a lot with adherence.” FG2  “And we would have more freedom.” FG9 |
| An app would not help in TB management, n=10 | “There are problems are so complex that they can’t be solved by eHealth.” FG9  “Make it harder, giving us the double job.” FG11 |
| **Code: eHealth would be faster, n=28** | |
| Key themes | Examples |
| An app would be faster, n=21 | “Regarding the studies, so now I don’t have to buy a guide, a printed guide, because I can download it from the internet, I save time, yes, I save a lot of time.” FG7  “We get results faster, like about lab tests, or about our disease.” FG5 |
| An app would be faster especially in regards to travel/distance, n=6 | “Yes because it would also help the people who live far away.” FG1  “I think it would ease. My home is far away, so if I come here I still need to queue, it will waste my time. If it is clear when is my turn, I can use my time for other things.” FG5 |
| Domain: Subjective norm  *How will others react? What are the societal/community norms concerning eHealth?* | |
| **Code: How will other people (the other group) react to an eHealth solution, n=52** | |
| Key themes | Examples |
| There will be challenges/ implementing the app will depend on certain factors, n=23 | “Simpler for the younger people, harder for the older.” FG9  “Mentality [will be a problem]. Scepticism before anything new. Something new we’re forced to do.” FG6  “If you were formed in a compute era, everything will be easier. We have examples here in our hospital, some people get it very quickly, some people not so much, because they don’t have the training, the experience with it.” FG6 |
| eHealth will be easily accepted, n=15 | “They will like it.” FG7  “I believe the staff will be open to this app because the workload will be easier to deal with.” FG1 |
| **Code: Communication – status quo, n=39** | |
| Key themes | Examples |
| Communication needs to be improved within our job/community, n=31 | “And we don't have a connection with labs. Results come on the post. We also don't have connection with the hospitals.” FG3  “People should tell us more things to our face. But also they should tell us the positive sides, not only the negatives. You know, one hot, one cold.” FG5  “And that is important time for the patient…if we had a rapid connection, we can have a communication with resistant tb specialist quicker and not waste the time of the patient.” FG8 |
| We have no problems communicating, n=6 | “We have never used internet to get In touch with doctors.” FG1 |
| Challenges in using an app to improve communication, n=2 | “Ok, trust in patient is very important and you have to have it, but you can't always be sure, he can always spit [the pills], you never know.” FG3 |
| **Code: Challenges to implementing eHealth within the local environment, n=38** | |
| Key themes | Examples |
| Infrastructure challenges: lack of network, physical, human resources, n=20 | “They get their phones stolen, or all sorts of cables can be robbed.” FG8  “The [clinic] does not have the manpower to see everyone, they go maybe once every two months, with this, you could see if someone has gained or lost weight.” FG9  “To add to that, I think that the use of data is one thing you should be looking at. It could be made in such a way that you download it as a one time download and probably there could be an update when necessary.”FG13 |
| Patient-related challenges, n=7 | “Right now we have mainly illiterate asylum seekers, no [nationals]” FG2  “Oh, this [an app with video-DOTS] would be great. But patients go work the fields, when would they do that?” FG6 |
| Medical staff-related challenges, n=7 | “I think the doctors don’t know about TB.” FG4  “They worked slowly though it was Emergency Room.” FG10 |
| Other challenges, n=3 | “There is no political willpower.” FG6 |
| No challenges perceived, n=1 | “Most of our patients are happy with the treatment and department.” FG2 |
| **Code: Information status quo, n=31** | |
| Key themes | Examples |
| Information is not accurate/ not enough, n=23 | “And also there is a huge lack of knowledge because for example I did the test I got the result with a positive cross and because I didn’t know what that medians I didn’t go to the hospital immediately.” FG7  “It’s sad to say but nowadays there is also a lot of ignorance among medical personnel related to the treatment of patients, especially those medical doctors who don’t work in public health.” FG8  “Well, sometimes you are searching for something for example in the internet, the websites that you researching, sometimes it delays. Sometimes the fault is from the website. Sometimes the delay is from the network. So if it is made easier for where we can find more information for TB.” FG12 |
| Information flow is imperfect, n=8 | “Information is good, but then there is too much information, you have to digest it. We have patients coming in, I know what I have. Let me test you versus the internet.” FG6  “What I know is what I’ve been doing in this project with asylum seekers and immigrants in [country], […] and from my experience is that there is a big difference between healthcare regulations in other countries and it's hard for this group of people to obtain accurate and fast information.” FG3 |
| **Code: Stigma and TB, n=28** | |
| Key themes | Examples |
| I felt stigmatised, n=27 | “People will say it to your face: <<move aside, TB-infected person!>>” FG5  “It’s enough to be a TB case in a school and everyone feels the stigma. It’s horrible.” FG6  “For example I feel a lot of rejection every time, people changed a lot when they found I am TB positive, they don’t even say hello directly to me, but from afar and this rejection is due to the lack of knowledge, education.” FG7  “I can say that maybe you go to any community with such a disease, some people will be afraid and think that you may infect them with the disease without knowing that I have taken the drugs for some time.”FG12 |
| I don’t feel TB is a stigmatising disease, n=1 | FG participant: “But we never had a problem with fear of transmitting it to people.”  Interviewer: “But other people were afraid?”  FG Participant: “No, we never had such problems.” FG1 |
| **Code: Other’s experience with tech, n=20 (Patients about medical staff and vice versa)** | |
| Key themes | Examples |
| I don’t think there will be problems in implementing a new eHealth app for the “other” group, n=12 | “I don’t think using an app will be a problem…even if they are illiterate they use smartphones…even if they illiterate they use the internet, google, even if they can’t write their own signature, they can go online.” FG2  “Yea, health application will be suitable for young people, since they stick to their gadgets.” FG10  “[People] You are aware that this thing [mobile apps] is happening and I have been seeing it on social media.” FG13 |
| I think there will be problems in implementing a new eHealth app for the “other” group, n=8 | “Our Tb patients are not there yet. We don’t really have university professors with TB.” FG6  “For example the people who don’t have resources they probably have 1 smartphone per family and they don’t use internet all the time.” FG7 |
| Domain: Perceived ease/difficulty towards behaviour  *Can I implement eHealth? Would it be easy or difficult? What would make implementation easier or more difficult?* | |
| **Code: I can/can’t use a new eHealth app, n=37** | |
| Key themes | Examples |
| I think I am capable of using eHealth in my work/daily life, n=15 | Interviewer: “How do you think adoption of a new app would be for you?”  FG participants: “Easy, easy.” [raised voice, altogether] FG7  “I don’t think it's going to be much of a problem.” FG13 |
| I am capable of using eHealth, if some conditions are met, n=16 | “It [the eHealth app] has to have more things to be usable or I use my regular things.” FG3  “And the time to learn it and the time you have to spend at work if you have your own patients there.” FG9  “Patient: I don’t think it will be a problem for me because it has become part of me. I have been using it. It is just a matter of maybe, for example upgrading an app, whether to access it.” FG12 |
| I don’t think I am capable of using eHealth, n=6 | “I am illiterate so I wouldn’t be able to use the app.” FG1  “And if you have too many apps, like we already do, to have another one, it could be time consuming. We wouldn’t want to be overloaded.” FG9 |
| **Code: Financial challenges/incentives, n=34** | |
| Key themes | Examples |
| In order to be able to implement an app, we need physical and human resources, n=16 | “We could give them the actual devices, like a smartphone.” FG2  “In my office I don’t have a go-to PC, nor a printer… the PC has a poor memory…so we then have to take the work home or go to another place to be able to do our job.” FG8 |
| The app should be free, n=7 | “If it’s free then we would definitely use it.” FG6 |
| There are some challenges concerning financial situations, n=8 | “We have a lot of immigrants and even [natives to the country] people, but homeless, with a lot of comorbidities, some even without a phone, but I think that for some it’s difficult to buy and use a phone…” FG9  “They [the patients] are poor.” FG6  “We don’t have money to buy credits, for the bundle and network.” FG12 |
| The app will be cost-effective, n=3 | “But it is cost effective long term.” FG2 |
| **Code: Community building features would help implement eHealth in TB, n=31** | |
| Key themes | Examples |
| A community building module for patients would/would not help in implementing eHealth, n=21 | “For example testimonials & interviews where people show they got treated & improved so they show the disease can be curable.” FG7  “Maybe new TB patients would benefit from old TB patients, how’s the food, what the nurses can do for you.” FG4  FG Participant no. 1: “Here, we talk all the time. I think it would be very useful.“  Interviewer: “What would you talk about?”  FG Participant no.2: “Ha, what and when was your last drink?” [laughter]  FG Participant no.1: “Pills, vodka, he, I finished treatment.”  FG Participant no.3: “This is a good way to establish relationships.” FG5 |
| An expert community would help in implementing eHealth, n=10 | “So to start contacting other peers, other specialists, to build up te information which will be managed among medical personnel and to also contact people and specialists from other countries.” FG8  “...and it would be good to work closer together because things would be easier.” FG3 |
| **Code: Privacy would ease behaviour, n=18** | |
| Key themes | Examples |
| Making sure the app is confidential should be a top priority/ would help in implementing eHealth, n=14 | “Privacy, that’s what I would consider first. There shouldn’t be any breaches because otherwise it wouldn’t succeed so no one should have access to data.” FG2  “The web page should be secure – we need internet security.” FG8 |
| I don’t care/ Confidentiality shouldn’t be a big problem, n=4 | “I would not care to show my identity as a TB patient to show that TB patients can survive.” FG7  “But it might not a problem as this is an app. We already had experience with [other apps], we have our own account, so only us who can see it.” FG11 |
| **Code: Videos would ease behaviour, n=18** | |
| Key themes | Examples |
| Educational videos would help eHealth adoption, n=12 | “But if there are videos… about how I should feel every week…first week this, second week [it would be useful].” FG4 |
| Video-DOT would help eHealth adoption, n=6 | FG participant no. 1: “…and DOT with video, and to call them to meetings.”  FG participant no. 2: “Real-time DOT?”  FG participant no. 3: “But maybe you could watch the videos in your own time  and the patient would be ok.”  FG participant no. 2: “Yeah you could walk and watch in between things.” FG3 |
| **Code: Training would ease behaviour, n=17** | |
| Key themes | Examples |
| Training would facilitate app implementation | “Training of the medical staff and the people who will actually administrate it.” FG2  “I think some education and orientation for users must be done, first there should be a socialization, then educate how to use the apps. Then make the e-manual.” FG10  “We would need a training module. For everyone.” FG6 |
| **Code: Localisation would ease behaviour, n=16** | |
| Key themes | Examples |
| Translation would help/should be a priority | “You get to work with different nationalities and every nationality has different backgrounds, so we need an app in different languages.” FG3  “And the language will be a problem, it has to be translated.” FG9 |
| Domain: Preferred Features  *What features or modules would I like the app to have?* | |
| **Code: Treatment module, n=45** | |
| Key themes | Examples |
| eHealth would help with appointments/ follow-up, n=12 | “and for making an appointment through mail or video” FG3 |
| eHealth would help with adherence/ video – DOTs, n=13 | “It [eHealth] would help a lot with adherence.” FG2  “and DOT with video” FG3 |
| eHealth would enable patients to have control over their own treatment through a dedicated log-in profile system, n=11 | “You can check your treatment so anyone with a phone can make use of this app.” FG7  “Yes, medical service through application. It also can be made separately, for doctor and patients.” FG10 |
| eHealth could help us monitor side effects, n=9 | “To monitor, we can monitor the patients easier, wherever they are” FG11  “So possible reactions as well as side effects of the medication should be looked out for.” FG13 |
| **Code: Media preference, n=38** | |
| Key themes | Examples |
| Video, n=18 | “I think film would be nice. When you read you can put it away.”FG4  “Educational videos.” FG8 |
| Images, n=12 | “Maybe some photos of the TST.” FG3 |
| Text, n=8 | “Text like in the language of the patient, yes!” FG3  “A message would be great. SMS.”FG6 |
| Audio, n=5 | “Audio would be best.” FG4 |
| **Code: Reminders, n=29** | |
| Key themes | Examples |
| A useful/the most useful feature in an app would be a reminder function, especially for treatment, n=24, or for appointments n=5 | “You need to make an app which tracks the pill box at the same time with an sms with a video. Though it’s still hard to be sure that he swallows his pills. Patients are very inventive.” FG6  “I say that the moment you receive the positive test they should alert you.” FG7  “Maybe reminders for contacts or for pills – and to also tell you that.” FG9 |
| **Code: User interface (UI), n=29** | |
| Key themes | Examples |
| UI should be simple/easy to use, n=14 | “It should be simple, with not many things, because it would demotivate me.” FG9  “But the application should be made as simple as possible.” FG11  "It depends on how the app is presented. I mean if I am trying to open this app and it says do this, open this site, do that, enter this, it becomes too complicated. But if the app itself is very simple to use, you open it and you are there: symptoms, you click there, complications, they are here, treatment model here, then it makes it simple. So I think it is how the app itself is presented to the people. If it is made simpler to use for everybody then it wouldn’t be a problem.” FG13 |
| UI should be friendly, n=5 | “You have to be very positive, not to scare them.” FG3  “No one wants to leave their comfort zone.” FG6 |
| UI – other recommendations, n=10 | “I think the design and options are important, and i think that the app could be a different way to work and think.” FG3  “Something striking, something that catches the attention.” FG8  “And something to help him make contact with you like a button with information about how to contact us.” FG9 |
| **Code: Prevention, n=22** | |
| Key themes | Examples |
| I would like an eHealth app to be used for educating for screening/prevention, n=17 | “Through this media/app patients can be educated and we can reinforce prevention.” FG8  “And also diagnostically because people don’t recognise the symptoms and if they were informed it would be easier.” FG2 |
| I would like an eHealth app to be used for prevention, n=6 | “I think the first one [feature to be implemented] should be prevention.” FG7 |
| **Code: Diagnosis, n=19** | |
| Key themes | Examples |
| I would like a diagnosis feature in the app, n=17 | “So the patients can also have the tools to self-diagnose.” FG8  “[I would like] monthly lab results.” FG8  “But also implement that the TB test should be the first exam done to diagnose.” FG7 |
| An app could never contain a diagnosis feature, n=2 | “But in no case it can replace the diagnostics of TB.” FG2 |
| **Code: Gamification, n=16** | |
| Key themes | Examples |
| Gamification would be a preferred feature, n=10 | “Or maybe a countdown, how far you are and how much you have left and then patients know what to eat. For them to see how easy it is and to stimulate them to continue.” FG3  “For example for younger, we need maybe little games.” FG9 |
| Within gamification, I think a quiz would be a useful module, n=6 | “Maybe some quizzes. This would be great. Anonymous, of course.” FG6 |
| **Code: Miscellaneous, n=10** | |
| Key themes | Examples |
| Miscellaneous features or ideas which would improve eHealth app or adoption | “Also if this could be known through the media and then it would get good feedback from the media and so it would be easier to implement because it would lead to actual awareness so more people would know about it and we could do it” FG2  “So, maybe psychological counselling? Morale is very low.” FG5  “Can you make it in offline mode first? Because not everyone has a good internet connection or data package. So, after download and we do not have internet connection we can still access the apps, can you do that?” FG10 |
| **Code: Repeatability, n=3** | |
| Key themes | Examples |
| Repeatability is a preferred feature | “You keep saying it, but you can replay a video online.” FG3 |
